# Illness experiences of diabetes in the context of malaria in settings experiencing double burden of disease in southeastern Tanzania

**DOI:** 10.1371/journal.pone.0178394

**Published:** 2017-05-25

**Authors:** Emmy Metta, Ajay Bailey, Flora Kessy, Eveline Geubbels, Hinke Haisma

**Affiliations:** 1 Ifakara Health Institute, Dar es Salaam, Tanzania; 2 Population Research Centre, Faculty of Spatial Sciences, University of Groningen, Groningen, The Netherlands; 3 Mzumbe University, Dar es Salaam, Tanzania; Public Library of Science, FRANCE

## Abstract

**Background:**

Tanzania is doubly burdened with both non-communicable and infectious diseases, but information on how Tanzanians experience the co-existence of these conditions is limited. Using Kleinman’s eight prompting questions the study synthesizes explanatory models from patients to describe common illness experiences of diabetes in a rural setting where malaria is the predominant health threat.

**Methods:**

We conducted 17 focus group discussions with adult members of the general community, diabetes patients, neighbours and relatives of diabetes patients to gain insight into shared experiences. To gain in-depth understanding of the individual illness experiences, we conducted 41 in-depth interviews with malaria or diabetes patients and family members of diabetes patients. The analysis followed grounded theory principles and the illness experiences were derived from the emerging themes.

**Results:**

The illness experiences showed that malaria and diabetes are both perceived to be severe and fatal conditions, but over the years people have learned to live with malaria and the condition is relatively manageable compared with diabetes. In contrast, diabetes was perceived as a relatively new disease, with serious life-long consequences. Uncertainty, fear of those consequences, and the increased risk for severe malaria and other illnesses impacted diabetes patients and their families’ illness experiences. Unpredictable ailments and loss of consciousness, memory, libido, and functional incapability were common problems reported by diabetes patients. These problems had an effect on their psychological and emotional health and limited their social life. Direct and indirect costs of illness pushed individuals and their families further into poverty and were more pronounced for diabetes patients.

**Conclusion:**

The illness experiences revealed both malaria and diabetes as distressing conditions, however, diabetes showed a higher level of stress because of its chronicity. Strategies for supporting social, emotional, and psychological well-being that build on the patient accounts are likely to improve illness experiences and quality of life for the chronically ill patient.

## Introduction

Most sub-Saharan African (SSA) countries are now doubly burdened in terms of disease, with non-communicable diseases (NCDs) emerging alongside the persisting infectious conditions [1]. While malaria, tuberculosis (TB) and acquired immune deficiency syndrome (AIDS) continue to threaten the population’s health in this region as they have for decades, other conditions such as diabetes, cardiovascular diseases, cancers and chronic obstructive respiratory diseases are now emerging, becoming major disease burdens in the region [2]. For example, in the year 2013, the African region experienced 128 million infections of malaria [3], and the number of people living with diabetes reached 19.8 million in the same year [4]. SSA countries account for 90 per cent of the global deaths due to malaria [3]; 8.6 per cent of all deaths among adults aged 20–79 years in the African region are attributed to diabetes [4].

Similar to the rest of Africa, infectious and non-communicable diseases coexist in Tanzania [5, 6]. Tanzania is the third on the list of the African countries with the highest population at risk of malaria after Nigeria and Democratic Republic of Congo [7]. Recent reports indicated a decline in malaria prevalence from 18 per cent in 2002/08 to 9.2 per cent in 2012 [8]. Indeed, despite a recent decline [9–11], malaria is still a major cause of illness in both children and adults in the country. About 10–12 million Tanzanians experience clinical malaria illnesses yearly, accounting for more than 40 per cent of the annual visits to public health facilities [12, 13]. Estimates suggest around 60,000–80,000 malaria deaths occur annually among all age groups in the Tanzania’s mainland [13]. Although children under five years remain the most susceptible age group to malaria, the recent literature shows that the level of malaria infection is shifting to the older age groups [14, 15] due to the intensification of the malaria control interventions and the population getting older. The observed changes in mosquito biting behaviour from indoor to outdoor biting [16] are also thought to increase adults’ susceptibility to the condition.

As communities are still struggling with the persisting infectious diseases, diabetes is becoming increasingly common, among other NCDs. The prevalence of diabetes in Tanzania has reached 9.1 per cent among adults aged 24–65 years and at roughly equal rates between men and women and in urban and rural areas [17]. Diabetes is one of the top ten leading causes of death in hospitals among Tanzanians aged 5 years and over [18], however, this can be an underestimation of the real contribution of diabetes to mortality rates, since only 1 in 16 deaths among Tanzanians age 5 years and over takes place in hospitals [18]. Population based surveys report that most people diagnosed with diabetes were not aware of their illness [19, 20]. In this context, therefore, malaria can be understood as an important cause of illness [10, 11] and diabetes as an emerging epidemic [21]; previously termed *“an epidemic in full flight”* [22], it is one of the major contributors to ill-health and premature death [21].

While malaria and diabetes have different aetiologies, meaning that they manifest differently and require different treatment and management models, it has been shown in other SSA countries that both illnesses occur in the same communities and sometimes co-exist in the same individuals and or households [23–26]. Literature shows that people with diabetes are at increased risk of severe malaria [23, 24], among other infectious and non-communicable diseases [27, 28]. There is also evidence that treating and managing malaria in people with diabetes is quite problematic [29] due to the effects of the malaria parasite and that of antimalarial treatments–especially quinine treatments on glucose homeostasis [23, 30]. The coexistence of these conditions adds stress to the already stressed individuals, families and the communities in general, in terms of the resource allocations for their management, and the increased burden of care to the households and their families. This in itself also increases the emotional, psychological and physiological pain of the sick individuals and their family members. Unlike many of the infectious diseases, NCDs such as diabetes, once manifest clinically, are not curable; rather, the patients have to live with the condition throughout their lifetime. Therefore, there is a need to document the lived illness experiences of NCDs in the context of existing infectious diseases, as these are not well understood. Such knowledge can be useful for practitioners and policy makers concerned with the management of these conditions for an enhanced quality of care of all people.

Understanding of the illness experiences requires looking into people’s narratives involving the illness–their illness explanatory model. The explanatory model of illness provides emic perspectives on how the patients’ illness experiences are shaped by their social and cultural context [31]. Explanatory model (EM) originates from the work of anthropologist Authur Kleinman [32], who was interested in understanding how chronic illnesses are experienced by the people. In his monograph, “*The illness narratives*,*”* Kleinman posits that when speaking of illness, we must include the patient’s judgments about how to best cope with the distress and with the practical problems it creates in daily living [31]. Kleinman [33] reasoned that health professionals need to understand how their patients conceptualize their illnesses in order to be able to provide effective care [33]. To understand patient illness experiences, Kleinman [33] recommended eight targeted questions as a framework for eliciting a patient explanatory model. Those questions included: (i) What do you call your problem? (ii) What do you think has caused your problem? (iii) Why do you think it started when it did? (iv)What do you think your sickness does to you? (v) How severe is your sickness? Will it have a short or long course? (vi)What do you fear most about your sickness? (vii) What are the chief problems your sickness has caused for you? And (viii) What kind of treatment do you think you should receive and what are the most important results do you hope to receive from this treatment? Although Kleinman’s EMs were originally developed for use by clinical and public health practitioners [23], they have been widely used for gaining insights into chronic disease aetiology [24, 25]; experiences with hypertension self-management [26]; understanding youth violence [27]; lay accounts of depression [28]; and cross-cultural conceptions of schizophrenia [25], to mention a few. In this study we have been inspired by the Kleinman’s eight prompting question to synthesize EMs from patients, and have generalized from these EMs to describe common illness experiences of diabetes in a context of malaria as a predominant condition.

According to Kleinman [31], illness is a social experience entailing the dynamic processes of how the patient and his/her family or social network perceive, live with and respond to symptoms and disability [31]. As such, the experience of illness signifies the subjective state of the sufferer in his/her social context. It involves the fluctuations in bodily functioning and how the disease is understood in ones’ daily life [34]. Whereas disease and illness are often used interchangeably [35], the two are not the same. Disease refers to the malfunctioning or maladaptation of biological and psychophysiological processes in the individual that may result in potential reduction of physical capabilities or of life expectancy [36]; illness, however, denotes the individual, relational and cultural reactions to the disease [37]. Illness is formed by the cultural characteristics that shape perceptions, classifications, descriptions, and evaluation of the upsetting experiences [31], a process that is embedded in the intricate interweaving of family, social and cultural ties. Therefore, the experiences of illness are better viewed from the perspective of illness than the perspective of disease because the latter perspective is not conducive to dealing with such personal, cultural and social aspects [37]. These concepts were applied in the study to elucidate the illness experiences of chronic NCDs in a geographical area dominated by acute infectious conditions.

Although illness is an inevitable fact of human life [38], illness experiences are discomforting and can interfere with many facets of life. The experience of being ill due to an acute infectious condition might not be the same as that of being ill with a chronic non-communicable condition, especially considering their differential nature in chronicity. Literature suggests that the illness experiences involving chronic conditions are surrounded by long-term courses, unpredictability of symptoms, disabling effects that are often accompanied by minimally effective treatments, social stigma, and isolation [39, 40]. Unrelenting illness uncertainties [41–43], limited social life [44], increased risk to infections [45] and functional limitations [46] are among the common experiences reported by people with chronic illnesses including diabetes. Insofar as an illness experience reflects a patient’s subjective viewpoint, it may thus bear limited relation to the clinical aspects of the disease. Therefore, illness experiences might have considerable implications for how people deal with the conditions, which in turn would impact the illness outcomes and social wellbeing.

The consequences of the coexistence of such detrimental diseases can be burdensome for the patients and their families. Living with a chronic, non-communicable illness is costly since once the disease manifests clinically, its treatments are expensive [47]. Chronic, non-communicable illnesses require management and continuity of care over many years, in contrast to most of the shorter-lived acute and infectious conditions. In low resource settings, households are sometimes forced to make difficult choices between getting medications for a chronically ill person and or getting relief for a household member suffering from an infectious disease [48]. Sometimes, the same choice has to be made for individuals who might be facing the two types of illness conditions simultaneously. This situation increases the burden of care for the families, perpetuates poverty and pushes people further into the vicious circle of poverty and diseases [49]. In reality, the double burden of disease not only challenges the household resources in responding to the conditions but also exposes patients and their families to continuous financial, social and emotional obligations regardless of their financial capability and social wellbeing.

Unfortunately, in-depth understanding of the illness experiences in the context of the double burden of disease in rural settings is limited, but such an understanding is crucial not only for targeted treatment and care, but also for informed decision-making and the designing of interventions for emotional and social support to patients for a better illness outcome. The current study uses Kleinman’s eight prompting questions on explanatory model of illness to elucidate the illness experiences on diabetes in the context of malaria from the participant’s own point of view. Studies using the explanatory model of illness experiences in rural settings are limited, particularly for SSA and Tanzania. The current study is part of a larger study designed to ascertain health-seeking behaviour practices among adults for malaria and or diabetes in rural communities of Tanzania. The results of this study will contribute to informing the design of context-adaptive intervention measures for disease prevention and management; they will also contribute towards the efforts to determine how best to address the personal, social and emotional challenges posed by the emerging NCDs in the context of the infectious diseases as predominant conditions.

## Materials and methods

### Study area and population

This study was conducted in Viwanjasitini and Namwawala villages and at the diabetes clinic in Kilombero district. Viwanjasitini and Namwawala villages (total population of 21,270) were purposively selected for the study based on their proximity to St. Francis referral hospital where the diabetes clinic is situated. This hospital provides various health services, including those for malaria. To allow the study to capture a wider range of the illness experiences on malaria and or diabetes, the study villages had both semi-urban and rural characteristics. The semi-urban village (Viwanjasitini) was located within 5 kms from St. Francis referral hospital, while the rural village (Namwawala) was 43 kms away from the hospital. The pilot that we conducted in preparation of this study clearly showed that people in the general community had limited real-life illness experiences with diabetes and the condition was considered relatively new compared to malaria. This meant that engaging general community members in focus group discussions on diabetes would not reveal any relevant substantial information about the shared illness experiences on the condition. The diabetes clinic, the only one in the district, was involved in the study to gain access to diabetes patients from the surrounding rural villages who were visiting the clinic for their monthly routine check-ups.

Malaria is endemic in Kilombero district [50, 51] and despite the recent recorded decrease [9], it remains a common cause of illness for people of all ages. The prevalence of malaria in the district is 14 per cent [52] higher than the national prevalence rate of 9.2 percent [8]. The climate of the district is conducive to both high and perennial malaria transmission [51, 53]. Alongside other predominant infectious conditions like human immunodeficiency virus (HIV) and AIDS [54] and tuberculosis [55], diabetes is of emerging concern in the district [20, 56]. The HIV prevalence rate in the district is 6.5 per cent and that of diabetes is 12 per cent [20]. Information on the true burden of TB in the district is lacking, however, there are reports that TB cases are on the rise throughout the area [57]. As the epidemiological transition progresses in this setting with chronic NCDs such as diabetes on the rise and alongside the higher prevalence of acute infectious conditions such as malaria, it provided the study with the opportunity to explore the illness experiences of a newly emerging non-communicable condition in an environment dominated by acute infectious conditions.

### Study design and methods

To explore collective illness experiences, we conducted 17 focus group discussions (FGDs) involving 117 adult community members between October and November, 2012. Among the participants, 59 were engaged in 8 FGDs (4 FGDs with men and 4 FGDs with women) that focused on the shared experience of malaria and included people from the general community. The remaining 58 participants were engaged in 9 FGDs (5 FGDs with women and 4 FGDs with men) that focused on the shared illness experiences of diabetes and they included diabetes patients and either neighbours or relatives of diabetes patients. Between February and March 2013, we conducted 41 in-depth interviews (IDIs). These IDIs focused on gaining deeper understanding of individual illness experiences and included 15 IDIs that focused on malaria and 19 IDIs that focused on diabetes. Seven other IDIs focused on getting insights on the issues raised by diabetes patients and engaged diabetes patient family members.

### Recruitment of the study participants

Participants on malaria FGDs from Viwanjasitini and Namwawala villages and on diabetes FGDs from Viwanjasitini village were purposively selected with the help of the village leaders. The malaria FGDs involved general community members and the diabetes FGDs involved neighbours or relatives of diabetes patients. Other FGDs on diabetes involved diabetes patients and these patients were purposively recruited from the diabetes clinic with the help of the clinic nurse.

The malaria IDIs were conducted with people who had recent malaria experience (14 days prior the interview date -15 IDIs) and were purposively recruited with the help of the village leaders in both Viwanjasitini and Namwawala villages. The diabetes IDIs involved diabetes patients with not less than six months since a diagnosis of diabetes. These participants were purposively recruited with the help of the village leaders (9 IDIs with diabetes patients in Viwanjasitini village) and the clinic nurse (10 IDIs with diabetes patients recruited through the diabetes clinic). Since the patient IDIs aimed at gaining insights into the lived illness experiences, a period of fourteen days with malaria and or six months with diabetes was considered enough for participants to have gained such experiences. Other IDIs (7 IDIs) involved diabetes patient family members who were purposively recruited with the help of the diabetes patients.

We visited households in the villages and asked about adult household members’ information on malaria and or diabetes illness experiences. We observed the treatments used and records related to the participants’ recent fever management to rule out non-malaria fevers. More than half of the 15 IDIs on malaria had their malaria confirmed via a blood slide. Sometimes we were referred to people who were believed to have diabetes in other households; we then visited those households and asked the same question. References of this kind facilitated the recruitment process, and were especially helpful given the lack of familiarity with the condition to the general community. To avoid recruiting people of the same social network, and to ensure that the study captured wider perspectives on illness experiences, different entry points were used. Once the individuals confirmed that they had diabetes, their clinic or medical cards were reviewed to confirm their diagnosis. After it was established that malaria and or diabetes were the causes of the illnesses experienced, individuals were given detailed information about the study and asked whether they were willing to participate. The diabetes IDI participants were asked to identify their family members or individuals who were taking care of them. Some of these individuals were purposively recruited to take part in the diabetes family IDIs. After providing consent, the FGDs in the villages (Viwanjasitini and Namwawala) were conducted at convenient places selected by the participants themselves, while the IDIs were conducted at the participants’ households. At the diabetes clinic, both IDIs and FGDs were conducted at a canteen near to the St. Francis referral hospital. When the FGDs and the IDIs yielded no new information at the level of specific disease and among both men and women, data saturation was confirmed; this determined the recruitment of the study participants.

### Data collection and analysis

The data collection team consisted of the first author and two research assistants with a social science background at postgraduate level who had had extensive experience in conducting qualitative research. Following the research training, the team piloted the data collection guides. The results from the pilot were used for refining the final guides before their use for data collection in the main study. The data collection took place in two separate rounds. The first round involved FGDs. The results from this round were used to sharpen the IDI topic guides that were used during the second round of data collection. The first author facilitated all the FGDs and the IDIs. In each of the FGDs, one of the assistants took notes. The group discussions lasted between one hour to one hour and a half while the in-depth interviews lasted between forty five minutes to one hour. All FGDs and IDIs were conducted in Swahili, a common language in the study setting and to the research team. The discussions and the interviews were digitally recorded with participants’ consent and transcribed verbatim within 48 hours of the time they were conducted. Only the research team had access to the information provided. All of the personal identifiers were removed from the data.

The transcripts were reviewed and cross-checked for accuracy by replaying the audio files while reading the transcripts by the first author before importing the transcripts into NVivo 9 (QSR International Pty Ltd, Australia), the programme used to facilitate the analysis. All the transcripts were analysed in their original language and only the quotations are translated into English. The analysis process had two levels. The first level of analysis involved developing inductive and deductive codes. Both the inductive and deductive codes were developed by the first author. These codes were shared and discussed among the first three and the last author. Discrepancies were minimal and were reconciled through discussions prior to the finalization of the codes, and to the coding of the data. After consensus on the codes was reached, the first author coded the data and the coding was reviewed several times by the second and the last authors. The data coding continued until no new codes emerged. The second level of analysis involved categorizing the codes into themes and family codes following principles of grounded theory. This process continued until no new themes or categories emerged. The validity and usefulness of the themes and the family codes were regularly assessed by the second and the last author to ensure consistency and coherency. This process was followed by the writing of the descriptive reports. The illness experiences were derived from the emerging themes.

### Ethical approval

The study was approved by the institutional review boards of the Faculty of Spatial Sciences, University of Groningen (RUG) in the Netherlands; the Ifakara Health Institute (IHI) in Tanzania (IHI/IRB/No. 19–2012); and the National Tanzanian Medical Research Co-coordinating Committee of the National Institute for Medical Research (NIMR/HQ/R.8a/Vol.1X/1389). All participants were thoroughly informed about the study and then verbal consent was requested before beginning each of the IDIs and FGDs. Considering the low-literacy setting of the research and to avoid rising tension and discomfort among study participants, verbal consent was the most suitable form of consent-taking. The consent was digitally recorded prior to the initiation of the interviews or discussions. This type of consent taking was approved by the Institutional Review Boards of IHI and RUG and the national ethics committee of NIMR. To ensure the anonymity of the study participants, all of their potential identifiers were removed from the data, and only their opinions are presented.

## Results

The study results are presented on some of the emerging themes via the Kleinman’s eight prompting questions as applied on diabetes. Those themes include: (i) illness severity and longevity, (ii) impact of the illness, (iii) psychological and emotional problems (iv) physical health problems, and (v) economic problems (see Table 1). These themes as linked to Kleinman’s framework of eliciting patients’ explanatory model of illness (see Table 1) were the main themes that shaped the illness experiences as embedded in their social and cultural context. The remaining themes–meaning giving to symptoms; causes; onset of the illness; fears about the sickness, and treatments and outcomes expected (see Table 1), have been presented elsewhere [56, 58].

**Table 1:** Kleinman’s questions and the emerging themes.

s/n	Kleinman’s questions	Emerging themes from the data
1	What do you call your problem?	Meaning given to the symptoms
2	What do you think has caused your problem?	Causes
3	Why do you think it started when it did?	The onset of the illness
4	What do you think your sickness does to you?	Impact of the illness
5	How severe is your sickness, will it have a short or long course?	Illness severity and longevity
6	What do you fear most about your sickness?	Fears about the sickness
7	What are the chief problems your sickness has caused for you?	Physical health problems, economic problems, psychological problems and emotional problems
8	What kind of treatment do you think you should receive and what are most important results you hope to receive from this treatment?	Treatments and outcomes expected

### Illness severity and longevity

Participants in both FGDs and IDIs expressed contrasting opinions regarding the severity and longevity of the conditions. Malaria was reported as a serious illness but acute and relatively shorter in duration. Conversely, diabetes was perceived as an emerging, life-threatening illness of great length and severity.

“*…this disease …is not like any other disease we know …it is a life-threatening disease…once you have “kisukari” that is all…you have to live with it throughout your life until when God wishes….there is nothing you can do to cure it.…*”

PID15D

Malaria was spontaneously expressed to have a shorter course than diabetes. Participants were of the opinion that it is easy for a patient to forget the sufferings from an ailment like malaria due to its shorter duration, but that was not the case for a severe, long-lasting condition like diabetes. Diabetes patients indicated that the obligation to take diabetes medication daily was a constant reminder of the severity and the longevity of the illness in their lives.

### The impact of the illness

The research setting has a long history of malaria and had benefited from more than 50 years of intensive malaria prevention and control activities on the part of the Ifakara Health Institute alongside the efforts of several other malaria control stakeholders in the area. The study findings suggested that this knowledge on malaria prevention and control has led to greater understanding of the disease, which in turn both shaped the illness experiences and was reflected in the patients’ health-seeking behaviour. Participants cited malaria as a fatal disease but one which could be successfully treated in a short period of time and in most cases people opted for self-medication and treatment, with consultations only made to medical experts when the disease symptoms worsened. Some participants reasoned that because of its prevalence, the condition had become a normal phenomenon:

“*…here in our place, we are living with it [malaria]… it is not that we hear of people suffering from malaria somewhere. No… we know it ourselves because we get sick from malaria so often that now it becomes a normal thing*”

FGDFM11

In general, the illness experiences of malaria suggest that people have learned to live with the condition and its impact is relatively bearable compared to diabetes. None of the study participants had experienced severe malaria. Diabetes, on the other hand, was indicated to cause serious life-long consequences to the patients and their families. The illness experience of diabetes was shaped by psychological and emotional, physical health and economic problems as consequences attributed to the condition.

### Psychological and emotional problems

Despite the perceived seriousness of malaria, the illness experiences on the condition were associated with less psychological and emotional problems compared with diabetes. The acuteness of the disease and the quick recovery experienced by the malaria patients could be a possible explanation of the illness experiences elucidated. The experiences of diabetes however, illustrated that the illness had contributed to psychological and emotional problems in patients’ daily life. In their accounts, diabetes patients reported that the illness had interfered with the ability to live well by putting them into a perpetual state of tension and uncertainty about their current and future life. This state of mind was often reported to escalate into anxiety among patients family members that the patient might die at any time:

“*…normally she [her daughter] doesn’t say anything to me but when she looks at me ….I just see she is very nervous….her eyes are full of fears and I know she is worried that I might die at any time.….she knows that with this diabetes “kisukari” life is not certain…*”

PIDI4D

The psychological consequences that diabetes patients and their families experienced were characterized by persisting depression, stress and tension. It was a common concern in the study that the disease made most of the patients’ lives miserable due to uncertainties surrounding their condition, and this misery extended to the family members and caregivers as well:

“*when you have a diabetes patient at home there is no happiness at all*, *you are stressed all the time… when you see the patient being a little more quiet than usual you start being sceptical/ uncertain … I don’t know*, *perhaps s/he is not feeling well*, *or I don’t know what … I mean*, *the patient is unhappy but the family members are even more so because they know with this patient if we just do some little thing wrong …anything can happen…*.*”*

FGDFD5

In elucidating the depressive situation and the worries that diabetes exerted upon the family, one of the family members said:

“*…although it is not only diabetes that is a life-threatening disease…diabetes is more shocking… you find the family is always stressed… To the extent that a person dares to think that our fellow [patient] here …might die any day ….we will not have him any longer… it really brings worries and grief, especially considering it is someone who is very dependable in the family*”

FMIDI6

The worries and grief of living with a patient ill from a chronic, lifelong disease such as diabetes can have potential psychological consequences for the family members. As one participant whose husband had diabetes and high blood pressure poignantly articulated:

“*I feel very sad really… I find it is as if this family is only me and my six children… his condition is undetermined … it is very much like being half-dead … as I am here… deep in my heart I know I am not with him anymore, as anything can happen at any time…*”

FMIDI7

It can also have negative effects with respect to the broader community, such as changing the individual/family identity and devaluing the patient by labelling him/her a useless member of the community; but these issues were not explored in the current study.

Some of the diabetes patients on insulin expressed concerns about what might happen to them with the recurring fainting they experience, especially if they have no one there to help. These patients reported to regain their consciousness state after being fed sugar syrup by their family members:

“*this issue of fainting is a little frustrating… I mean it is difficult to understand and describe but it has happened to me several times …these days I am getting worried as to where the disease is taking me…. and what if she [wife] is not around?*”

PTIDI10D

Emotional problems were another characteristic that shaped the illness experiences of diabetes. The emotional problems were indicated to be a consequence of the patients’ failure to provide for their own and of their dependency on their families and relatives. This situation made some of the patients regard themselves as becoming a burden on their families:

“*… sometimes I feel sad, asking help from others. I am always asking help from my husband and daughter, and even though they don’t seem to mind helping me, I feel dejected. I feel like am becoming a burden on them…*”

PTIDI7D

The majority of the diabetes patients reported becoming too emotional and getting quick tempered as a new experience since the diagnosis, exhibiting something unfamiliar to their pre-diabetes personality. To lessen conflict possibilities within their families, some of the participants reported informing their family members explicitly about their personality change:

“*I told everyone in the family about my condition…They need to accept me as I am, I get angry faster now and sometimes I fail to control myself …I am thankful to God that they understood and are tolerating my condition…*”

PTIDI3D

### Physical health problems

Unlike malaria, the accounts of diabetes suggested that diabetes weakens patients’ systems and makes them more susceptible to malaria episodes and to other illnesses. Some participants in this study had diabetes and other chronic conditions like blood pressure, heart problems and strokes. In both FGDs and IDIs, participants claimed that diabetes made most of the patients get ill frequently and in most cases their illness conditions became seriously severe. Some diabetes patients mentioned frequent and severe malaria attacks:

“*…I get malaria frequently…and because of this sugar, the malaria becomes so severe–maybe because my body is already weak…*”

PTIDI2D

Another of the family members said:

*“This whole month he* [the diabetes patient] *has been in the house*, *just sleeping …*.*he was complaining of pains all over the body*, *dizziness*, *numbness and burning legs and arms … he could not get outside*, *everything was inside*. *I had to put a tin for him to urinate there… I mean it is a problem because he gets sick pretty often…*. *it has been like every disease is his now …”*

FMIDI4

Female diabetes patients voiced their concerns on the frequency of yeast infections they experience:

“*…Frequently I get yeast infections and that thing itches a lot…*”

PTIDI3D

The risk of frequent bouts of malaria and other diseases as a physical health problem exacerbated by diabetes can negatively impact not only the patients’ health but also the social and the psychological wellbeing of their family members.

While elaborating on the chief problems the illness has caused, most diabetes patients reported that diabetes made their health condition unpredictable and challenging most of the time. Many of these patients claimed that “the condition of the diabetes patient’s health could change at any time”–having good and bad days were thus common experiences with diabetes. Good days were those where patients experienced fewer symptoms, and bad ones were those days where patients experienced more health problems and became weak/sick. Diabetes patients complained about this unpredictability of their condition as preventing them from being able to plan in advance and organize their days:

“*…with diabetes you cannot know how it is going to be and plan your day… because sometimes you feel better and can work well but other times you become unable even to help yourself*”

*PTIDI1D*.

The unpredictability of the diabetes patient’s health condition was indicated to contribute to their failure to make joint activity plans with their family members. This was also shown to contribute to some community members’ doubts about diabetes patients’ physical health problems, as most of the time these patients did not have any observable symptoms.

Other patients complained that diabetes triggered the abrupt loss of memory and or poor concentration:

“*It happens that you sometimes forget even what you are doing…. You may be talking to someone but after few hours you don’t even remember having met …*”

PTIDI16D

Some diabetes patients reported decreased sexual desire/libido as a physical health problem they experience with diabetes. Some male patients affirmed to have experienced erection problems shortly after they were diagnosed with diabetes.

*“When this disease started*, *it was a little better … but then it did not take time…*.*it became completely impossible … whatever I do*, *that thing* [penis] *fails … it does not come up … I mean*, *I cannot do anything* [cannot perform sex]*…”*

PTIDI18D

Complaints of dryness and lack of interest in sex were expressed by female diabetes patients. These patients were of the opinion that with their diabetes health problem, staying married required them to have a husband with a great heart:

“*With diabetes your husband needs to have a great heart…*.*he has to understand …*.*in marriage… you cannot perform*…, *you fail completely to satisfy him… I mean you don’t feel it…actually it becomes very dry and you hate doing that thing… if the person does not understand*, *he may divorce you…*.*”*

PTIDI3D

The effect of diabetes on patients’ sexual desire/ libido can thus contribute to potential family problems such as separation or divorce and frequent misunderstandings and arguments; however, this would clearly need further exploration.

### Economic problems

All participants reported that illnesses either of malaria or diabetes caused economic problems to the patients and their family, but these issues were more pronounced for diabetes because of the recurrent costs involved. The cost of treating a single malaria episode for an adult in the study district using the recommended anti-malaria medicines could be Tsh. 1500/ = (USD 0.96), while a monthly medication for diabetes patients could range from Tsh. 4600 to Tsh. 52000 (USD 2.9–33.7), depending on whether one uses oral hypoglycaemic medication or insulin, respectively. The continuity of care required for diabetes and the related costs were often reported to have exhausted not only the patients’ savings but also that of their families.

Most of the patients claimed that diabetes contributed to their loss of income, as it prevented them from engaging in income-generating activities. They emphasized that the failure to earn an income was not only a problem in managing their daily life and affording their medications, but it also made them completely dependent on their family members:

“*Before I got this disease I was earning an income… I was working as a driver…. but ….since I got this disease that was the end of me…. I became completely dependent and my life has been horrible… getting food here is a big problem … I have to wait for the children to provide…. my main job has been just sitting at home… I cannot do anything…this is real a problem…*”

PTIDI10D

The accounts on diabetes suggested that because of the condition, some of the diabetes patients family members refrained from engaging in income-generating activities in order to be available to take care of them. They then indicated that this worsened the family economic situation. Such circumstances were especially severe for the families who started off poor even before they had a diabetes patient.

“*… when I was very sick my wife had to stop her business to take care of me…and because of the real situation she used the capital to pay for the bills…. since then she has never been able to go back … That is why I say this Kisukari has brought poverty upon us and there is no hope that this situation will change.…*”

PTIDI119D

Most of the family members complained that they had frequently reallocated their family income to meet the requirements of the diabetes patient and the related expenditures. This situation reportedly frustrated the family’s economy and their plan for development activities:

“*Honestly, you get to a point where you completely fail to invest in other things,…simply because of the need to take care of this patient.….sometimes you plan that you will do this and that for your own development but unluckily, you suddenly find her condition is not good …then you become forced to stop your other plans and take care of her because you cannot leave the patient just like that… as a result, you find that ultimately all the money is going towards her care…*”

FMIDI2

Some of the diabetes patients in both FGDs and IDIs asserted that, because of their loss of income, in most cases, their families were forced to sacrifice other household necessities such as house renovation, sending children to school, and buying clothes to be able to take care of their illness.

Female diabetes patients reported that diabetes affected their functional capability, making it difficult to perform not only income-generating activities but also their household responsibilities:

“*Sometimes even washing and cooking at home happens to be difficult. I feel ill and weak all the time and my body is weaker. So I just lie down a lot waiting for my daughter to help me with all that…*”

PTIDI13D

These concerns were reflected in the discussions with the diabetes patients’ family members, who saw diabetes as a strange illness that could cause functional incapability:

“*…she is not able to do anything tough …I mean she’s turned into someone we have to look after like a child… you have to find food and cook for her … only on rare occasions can she cook for herself but you need also to make sure everything is closer to her …. Honestly, this is a very strange/bad illness… it can make a person completely disabled*”

FMIDI2

The prolonged illness and the increased functional incapability caused by diabetes put pressure on other family members, who, in addition to performing their routine activities, also had to unceasingly assume the responsibilities of the sick individuals.

### Illness coping strategies

The study revealed that patients are not passive agents; rather, their illness experiences shape their self-perception, their evaluation and interpretation of symptoms, and their coping strategies. Diabetes patients indicated modifying their pre-existing behaviour–thereby shaping what we have termed their ‘illness coping strategies’–to accommodate social expectations, their own expectations and their current health status.

In both FGDs and IDIs, participants reported that diabetes made patients monitor their bodies, which is essentially specific illness coping behaviour. Diabetes patients in the study reported modifying their eating habits by becoming conscious not only of what to eat and drink; but also of the amount and the combination. Some of these patients claimed to have experienced difficulties in refraining from eating their traditional diet–which they referred to as “good food”. Despite that, they were obliged to follow the recommended modified diet. For example, most of the patients claimed to consistently avoid eating sugary foods so as to limit the rise of their blood sugar levels:

“*…the main challenge I see is getting used to the food …myself, I have diabetes and BP. I was advised to stop salted and sugary foods …so you can imagine this is something that I had never thought about… but I had no choice……at first it was really difficult; I could not manage it and as a consequence I was always sick … the sugar was very unstable and the BP was rising all the time…*”

PTIDI5D

Some diabetes patients reported challenges with their household budgeting because they not only had to budget for the family food but also for the costly recommended diet suitable for diabetes patients:

“*The fact that we have to differentiate my food from that of the rest of the family is already a challenge on our budget on food. It means not only cooking two pots; also, the type of food that I am recommended to use is not easily accessible and again it is very expensive compared to that of the rest…*”

PTIDI2D

The above excerpt indicates that meeting the recommended modified diet for diabetes patients not only challenged the families’ ability to acquire the foods but also to prepare them. This suggests that the family members responsible for preparing the meals needed to allocate more time to manage the cooking for the family and also accommodate the patient’s needs.

Limited social life was indicated in the study as a common experience among diabetes patients. Most of these patients reported being unable to engage actively in social gatherings and parties due to their restricted diet, fatigue, and the frequency of having to urinate. This practice may contribute to group isolation for diabetes patients in the community. Despite malaria being highly prevalent in the community and actually affecting people possibly more than any other disease, participants reported malaria as causing them less restriction, shame and stigma problems. This could partly be explained by the wider knowledge and the experience involving malaria that people have acquired over time–they have learned to live with the condition. The association of malaria to external causal agents–“mosquitoes”–might also have made the condition more socially acceptable.

## Discussion

This qualitative study provides important insights into illness experiences in the context of the double burden of disease. Malaria and diabetes were perceived as distressing diseases in this research setting, similar to other SSA countries burdened by a prevalence of both infectious and chronic diseases [21, 49]. As both of these conditions are present in the country, it is not surprising to find them co-existing in a single patient. The diabetes patients voiced concerns regarding the frequency and severity of malaria episodes they experience. The likelihood of diabetes patients being susceptible to severe malaria infections has also been reported elsewhere [23, 24].

Although malaria and diabetes are treated and managed differently, the illness experiences of the diabetes patients suffering from malaria have been reported as particularly challenging [23]. Quinine treatments—one of the most efficacious anti-malaria treatments—are known to induce a release of insulin in the body, leading to hypoglycaemia [59]. As a consequence, the use of quinine in treating malaria on insulin-dependent diabetes patients is highly risky [29]. It has been shown that the effects of the falciparum malaria parasites and that of the antimalarial quinine on glucose homeostasis complicates the treatment processes in patients with co-existing conditions of diabetes and malaria [23, 30]. The chronicity and severity of the diabetes patient’s condition dramatically impacts the family members and the frustration they experience around living with and caring for the patient can be as debilitating as the illness itself. This finding suggests that health practitioners need to counsel diabetes patients and their family members about the consequences of malaria–namely that it can be more severe for diabetes patients than for non-diabetes patients.

In contrast to malaria, the accounts of diabetes were associated with the patients’ increased risk to other illnesses, both infectious diseases and NCDs. The association of diabetes and the increased risk to contract infectious diseases such as pneumonia [60], bacteraemia [61, 62], tuberculosis [27] and non-communicable conditions like cardiovascular disease [28] and renal disease [63] is well documented. Recent literature, including systematic reviews and meta-analysis, reported diabetes doubles or triples the risk of patient’s developing active tuberculosis [27, 64–66]. In other parts of Tanzania, diabetes has been reported as a risk factor for pulmonary tuberculosis [67] and a stronger predictor of mortality during tuberculosis treatments [68]. The patients’ increased risk of contracting other illnesses could mean more suffering for the patient’s family in terms of the costs involved, social and the psychological pains of consistently caring for the sick. Medical professionals need to inform diabetes patients and their family members about the patients’ high risk level with respect to other illnesses. Our findings suggest that preventing diabetes and its complications will save patients and their families a lot of stress [69]. However, because the development of diabetes is insidious, strategies for its effective prevention should reach into community life well before the individuals in the community realize they are ill or even vulnerable to diabetes. This also goes for all NCDs, and would require not only economic resources but political backing.

The illness experiences of diabetes ranged from the unpredictability of the patients’ health to uncertainty about the patients’ day to day life because of not knowing what the following day would bring. Unpredictable health and uncertainty about the future has been described as a defining feature of people with chronic illnesses [42, 43, 70]. In this study, diabetes patients indicated a failure to plan in advance and organize their days due to the unpredictability of their health condition. Aujoula and colleagues [41] similarly reported that the majority of the people with cancer, asthma and diabetes experienced difficulties in planning for both short- and long-term projects because of their uncertainty regarding the future course of their illness. Life uncertainty due to unpredictable health problems can lead to a loss of hope for the patients’ survival, which consequently impacts the patients’ health outcomes as well as his/her family’s efforts against the illness. Medical practitioners could elicit discussion about the uncertainties associated with their patients’ condition and help them to identify coping strategies, thus contributing to the improvement of their emotional, social and psychological well-being.

The accounts of diabetes revealed patients’ decreased sexual desire accompanied by performance problems. Sexual dysfunction and its related psychological effects have been reported as health problems to pay attention to when aiming at improving the psychological well-being and the quality of life of diabetes patients [71]. Erectile health problems have been negatively associated with a range of psychological issues [71] such as severe depression, increased anxiety and poor self-esteem [72]. Sexual dysfunction has been recognized as a serious health problem among diabetes patients but has so far received little attention [72]. Decreased sexual desire among diabetes patients can negatively interfere with personal relations in their families as well as their identity within the community. The findings of this study suggest that establishing patient-centred interventions on psychosexual and relationship counselling among diabetes patients could improve their psychological well-being and health outcomes in general.

In the study, diabetes was associated with a more prolonged functional incapability than was the case for malaria. Diabetes patients reported that the inability to function and attend to their responsibilities was triggered by bodily weakness and exhaustion due to their diabetes. Functional incapability has been reported as a common health problem among people suffering from chronic illnesses [73] and diabetes in particular [46]. The experiences of functional incapability in this study made some patients see themselves as a burden on their families. The feelings associated with “becoming a burden” can impede patients’ agency with respect to their own life, as well as raise negative attitudes or habits of self-deprecation that can be depressing to the caregivers/family members who are trying to be supportive and understanding. There is an urgent need for interventions to delay or prevent the health problems associated with functional incapability among diabetes patients.

Participants expressed that illnesses are costly experiences, infringing not only upon the patients’ economy but also that of their family, albeit more so for chronically ill patients. Chronic non-communicable diseases like diabetes, once established, limit the productivity of most of the patients and their family members over the remaining time of the patient’s life, which puts great strain on the family economy. These economic problems have been reported as being overwhelming [74]. In the current study, the family members’ intermittent withdrawal from income-generating activities to take care of the diabetes patient seriously contributed to their family economic problems. The prioritizing of the patients’ needs over other household necessities contributed to complicating the family’s economic situation. It is important to ensure that people with chronic NCDs such as diabetes are treated properly, although costly, to at least be able to remain socially and economically active.

Emotional problems related to feelings of helplessness and being reliant on others, as well as quick tempers and a limited social life, appeared to shaped diabetes patients’ accounts in this study. In contrast to malaria patients, diabetes patients reported a failure to engage in regular social activities due to the frequency of needing to urinate, fatigue and the restrictions imposed on their diet. These study results are in line with observations made elsewhere on diabetes and other chronic illnesses [44]. The emotional issues of diabetes patients and the related failure to participate in social activities may lead to stigma and social isolation in the community. Social isolation has been reported to contribute negatively to physical and psychological health [75]. Stigma can have negative consequences on different dimensions of the disease like the illness experiences, care-seeking and limited supportive services from within and outside the families. Public health practitioners could consider counselling patients on the emotional and psychological aspects of the disease; to elicit their concerns and facilitate the development of coping strategies to adjust to their condition.

Participants in the study believed that sugary foods contributed to the cause of diabetes and or its related complications. Similar observations were also made in another east African country [76]. The association between excessive sugar intake and development of chronic conditions such as diabetes is well documented [77]. These study findings can benefit public health professionals in the design of behaviour-change interventions such as nutritional and dietary programmes in the community [78].

Explanatory model is a useful theoretical framework for understanding how illness experiences are embedded in the context of the individuals’ lives [33, 79]. The use of the Kleinman’s eight prompting question to synthesize EMs from patients facilitated the study to shed light on chronic non-communicable disease experiences in communities dominated by acute infectious conditions. The data gained in this research provide a unique, socio-cultural understanding of diabetes patients’ illness experiences and the meanings they create in their day-to-day living with the disease. These findings provide a better understanding of the decisions that patients make about their illness as well as their behaviour; this research thus illuminates the importance of considering patients’ explanatory model of illness experiences, especially in provisions of health services for improved health outcomes. Whereas disease perspectives are better viewed from the health professionals’ EM, the subjective experiences of the conditions are better viewed from the patients’ EM. Negotiations between these two models would help improve care and contribute to a better understanding between patient and health professionals. The study findings can contribute to informed decision making for public health practitioners, policy makers and those concerned with devising mechanisms for enhancing quality of care and improved well-being of all people.

Although most participants in the study, including diabetes patients, had personally experienced several bouts of malaria in their lifetime, some of the participants had no real-life illness experience with diabetes. However, the inclusion of the non-diabetes participants in the study was critical for a deeper understanding of how the condition is seen and experienced in the community. Conducting this study in a setting with a long history of malaria, a district that had therefore experienced intensive malaria prevention and control interventions, was vital in understanding how the illness experiences of the emerging conditions are framed in the context of familiar diseases like malaria. Although generalization of the findings should be approached with caution, the illnesses experiences detailed in the study are most likely to be similar in other settings because the predominance of malaria and the emergence of diabetes is a shared theme throughout Tanzania as well as the rest of SSA [1, 2, 21]. The study findings can provide us with a dynamic view of the social dimensions of illness and the related behaviours that illness precipitates.

## Conclusion and recommendations

The accounts we elicited in this study revealed that illness is a distressing experience regardless of whether it is malaria or diabetes. However, the experiences on diabetes seemed to portray a more severe picture than those of malaria because of the severity and longevity of the condition after its clinical manifestation. Unpredictability of health conditions, decreased sexual desire, and functional incapability were physical health problems pronounced more for diabetes than for malaria patients. Feelings of helplessness, quick temper and limited social life were common illness experiences among diabetes patients. Health-care practitioners need to consider the patients’ accounts of their illnesses if they are to provide care that is responsive to the patients’ needs and that helps them to cope with their illnesses for improved health outcomes.

Strategies building on patients’ accounts to design interventions to support their social, emotional, and psychological well-being are likely to improve the illness experiences and quality of life for the chronically ill. An assessment of the interlinkage between malaria and diabetes in terms of the meanings associated with the symptoms, their physiological and psychological aspects could provide more evidence on how these conditions and their treatments influence each other. Such information could yield useful insights for the public health arena and could contribute to shaping support programmes to lessen the consequences of the disease(s) on the patients and their families.

## Supporting information

S1 Data (DOC)
