# Supplementary material for: Illness experiences of diabetes in the context of malaria in settings experiencing double burden of disease in southeastern Tanzania
Source: PLoS One. 2017 May 25;12(5):e0178394. doi: 10.1371/journal.pone.0178394 (PMC5444834; doi:10.1371/journal.pone.0178394)
Supplement: S1 Data — (DOC) [file pone.0178394.s001.doc]

**Illness experiences of diabetes in the context of malaria in settings experiencing double burden of disease in Southeastern Tanzania**

**Minimal data set that forms the basis of the findings presented under the results section**

| **Source** | **Swahili version** | **Translated English version** |
| --- | --- | --- |
| FGDFM11 | Yaani sisi hapa kwetu tunaishi nayo ee bwana sio kama tunasikia watu wanaugua malaria sehemu fulaani hapana hiyo sisi wenyewe tunajua sababu tunaumwa malaria kila maara na ugonjwa huu kwa sasa umekuwa tu kama kitu cha kawaida | …here in our place, we are living with it [malaria]… it is not that we hear of people suffering from malaria somewhere. No… we know it ourselves because we get sick from malaria so often that now it becomes a normal thing |
| FGDFD5 | Unapokuwa na mgonjwa wa kisukari nyumbani hakuna raha kabisa (mmh) wakati wote mnakuwa na wasiwasi tu eeh yaani hakuna raha mama sababu unapomwona mgonjwa amenyamaza kidogo tu tofauti na kawaida yake wewe huku unaanza kuhisi (eehee) sasa sijui nini tena eeh labda hajisikii vizuri au sijui kafanyaje yaani nina maana jinsi mgojwa yule asivyokuwa na raha basi wanafamilia ndio kabisa hamna raha kwa sababu wanajua kwa mgonjwa huyu tukikosea kidogo tu basi chochote kinaweza kutokea | when you have a diabetes patient at home there is no happiness at all, you are stressed all the time… when you see the patient being a little more quiet than usual you start being sceptical/uncertain … I don’t know, perhaps s/he is not feeling well, or I don’t know what … I mean the way the patient is unhappy it is even more so for the family members because they know with this patient if we just do some little thing wrong …anything can happen…. |
| FMIDI4 | Mwezi huu wote ameshinda tu ndani analala (mmh) yaani yeye analalamika maumivu mwili wote, mara kizunguzungu, ganzi na hiyo miguu na mikono inapata moto (mmh) anasema huku eeh huku kote huku (anaonyesha miguu na mikono), hakuweza kutoka nje kabisa kila kitu ni ndani na ilibidi nimwekee kopo la kukojulea huko aaah ni shida (mmh) yaani ni shida kwa sababu anaugua mara kwa mara siku hizi mpaka hata sijui ufanyaje yaani imekuwa kama magonjwa yote ni yake sasa…… | This whole month he [the diabetes patient] has been in the house, just sleeping ….he was complaining of pains all over the body, dizziness, numbness and burning legs and arms … he could not get outside everything was inside; I had to put a tin for him to urinate there… I mean it is a problem because he gets sick pretty often…. it has been like every disease is his now … |
| PIDI5D | ……. huu ugonjwa sio kama ugonjwa mwingine wowote ule tunaoujua kwa mfano (mmh) ni ugonjwa unaotishia maisha yaani unapokuwa tu na kisukari yaani wewe ni basi (kwa nini?) ni lazima uishi nacho maisha yako yote mpaka Mungu atakapopenda (mmh) hakuna kitu unachoweza kufanya kuponyesha huu ugonjwa | ….this disease …is not like any other disease we know….it is a life-threatening disease …once you have “kisukari” that is all…you have to live with it throughout your life until when God wishes….there is nothing you can do to cure it…. |
| PIDI4D | ……kwa kawaida haniambii chochote lakini anaponitazama tu mie naona tu hapana anamawazo na wasiwasi mkubwa (kwa nini?) sababu macho yake yamejaa hofu kubwa na ninajua anaogopa labda naweza kufa wakati wowote (mmh) sababu anajua huu ugonjwa wa kisukari maisha hayatabiriki | …normally she [her daughter] doesn’t say anything to me but when she looks at me ….I just see she is very nervous….her eyes are full of fears and I know she is worried that I might die at any time.....she knows that with this diabetes “kisukari” life is not certain… |
| PTIDI2D | Yaani mimi napata malaria mara kwa mara na kutokana na hiki kisukari malaria inakuwa kali sana (mmh kwa nini inakuzidia) sijui inawezekana sababu mwili wangu umeshakuwa dhaifu | …I get malaria frequently…and because of this sugar, the malaria becomes so severe – maybe because my body is already weak...” |
| FMIDI6 | Kweli tunasikitika sana ingawa sio ugonjwa wa kisukari tu unaotishia maisha lakini huu unatisha zaidi (kwa nini?) yaani unakuta kila wakati famia inawaza (mmh) mpaka inafikia hatua ya mtu kufikiri kwamba mwenzetu huyu hali yake hii anaweza kufa siku yoyote (mmh) hatutakuwa naye tena (mmh) yaani ugonjwa huu unaleta wasiwasi na uchungu mkubwa hasa ukizingatia mgojwa mwenyewe ni mtu ambaye anategemewa sana na familia | “…although it is not only diabetes that is a life-threatening disease…diabetes is more shocking… you find the family is always stressed… To the extent that a person dares to think that our fellow here [patient] …might die any day ….we will not have him any longer… it really brings worries and grief, especially considering it is someone who is very dependable in the family |
| FMIDI7 | Mimi ninajisikia vibaya mno kwa kweli (mmmh) yaani ninakuta kama vile sasa hii familia ni mimi tu na hawa watoto wangu sita (mmh) maana hali yake haitabiriki kusema kweli ni kama tu mfu nusu (mmh) yaani hapa nilipo mimi kwa sasa nasema ndani ya moyo wangu najua kabisa siko naye tena sababu chochote kinaweza kutokea wakati wowote ….. | I feel very sad really… I find it is as if this family is only me and my six children… his condition is undetermined … it is very much like being half-dead … as I am here… deep in my heart I know I am not with him anymore, as anything can happen at any time...” |
| PTIDI3D | Huu ugonjwa mimi umenifanya napata fungus huku chini mara nyingi na hii kitu inawasha kweli kweli (mmh) nikienda hospitali wananiambia ni kwa sababu ya hiki kisukari | …..Frequently I get yeast infections and that thing itches a lot… |
| PTIDI1D | … na hiki kisukari huwezi kujua itakuwaje ili upangilie siku yako (mmh) maana wakati mwingine unajisikia vizuri na unaweza kufanya kazi bila shida lakni saa ingine unakuwa huwezi hata kujisaidia wewe mwenyewe | ….with diabetes you cannot know how it is going to be and plan your day… because sometimes you feel better and can work well but other times you become unable even to help yourself |
| PTIDI16D | Inatokea wakati mwingine unasahau hata unafanya nini eeh (kivipi yaani) unaweza kuwa labda unaongea na mtu lakini baada ya nasaa machache tu hivi hukumbi hata kama ulishaonana na huyo mtu | It happens that you sometimes forget even what you are doing…. You may be talking to someone but after few hours you don’t even remember having met … |
| PTIDI13D | Wakati mwingine hata kupika tu na kuosha vyombo inakuna kuwa vigumu (mmh) yaani najisikia kuumwa na dhaifu wakati wote yaani na mwili wangu sasa ni dhaifu sana (mmh) kwa hiyo mara nyingi nalala tu nikimsubiri binti yangu anisaidie kufanya vitu vyote maana mie siwezi tena | “Sometimes even washing and cooking at home happens to be difficult. I feel ill and weak all the time and my body is weaker. So I just lie down a lot waiting for my daughter to help me with all that …” |
| PTIDI10D | Hili swala la kuzimia linanichanganya kidogo sababu yaani ni ngumu kuelewa na kulielezea ila tu ni kitu ambacho kimenitokea mara nyingi sana (mmh) na siku hizi nimeanza kuwa na hofu (mmh) eeeh maana huu ugonjwa unanipeleka wapi hebu niambie (mmh) na sasa itakuwaje kama huyu mama hayupo karibu? karibu ….. | “this issue of fainting is a little frustrating… I mean it is difficult to understand and describe but it has happened to me several times …these days I am getting worried as to where the disease is taking me…. and what if she [wife] is not around? |
| PTIDI10D | Kabla sijapata huu ugonjwa nilikuwa na kipato changu tu kizuri (mmh) mimi nilikuwa nafanya kazi kama dereva wa haya magari makubwa (mmh) lakini tangu nilipopata hiki kizukari ndio ulikuwa mwisho wangu wa kazi (mmh) yaani nimekuwa tegemezi asilimia mia na maisha yangu ni matatizo matupu nakuambia (mmh) yaani kupata tu chakula hapa ni shida sana (mmh) inabidi tu nisubiri watoto ndio wanipe kila kitu (mmh) kazi yangu imekuwa tu ya kukaa nyumbani maana siwezi kufanya chochote (mmh) hii kweli ni shida jamani | Before I got this disease I was earning an income… I was working as a driver…. but ….since I got this disease that was the end of me…. I became completely dependent and my life has been horrible… getting food here is a big problem … I have to wait for the children to provide…. my main job has been just sitting at home… I cannot do anything…this is real a problem |
| PTIDI3D | Ukiwa na kisukari mme wako inabidi awe na moyo sana maana inabidi aelewe kabisa sababu kwenye ndoa inakuwa ngumu (mmh) huwezi kufanya kabisa yaani unashindwa kabisa huwezi kuridhisha hata kidogo (mmh) yaani unakuwa hujisikii kile kitu (mmh) yaani kwanza unakuwa mkavu sana na hupendi kufanya yale mambo ndio maana nasema kama mtu anakuwa sio muelewa anaweza kukupa talaka …… | “With diabetes your husband needs to have a great heart….he has to understand ….in marriage… you cannot perform..., you fail completely to satisfy him… I mean you don’t feel it…actually it becomes very dry and you hate doing that thing… if the person does not understand, he may divorce you….” |
| PTIDI2D | Ile tu kwamba inabidi tutenganishe chakula changu na cha wengine kwenye familia tayari ni changamoto kwenye budget ya chakula. Hii inamaana sio tu sababu inabidi tupike sufuria mbili lakini pia kwa sababu aina ya chakula ninachoshauriwa kula hakipatikani kwa urahisi na tena ni gharama sana ukilinganisha na chakula cha wanafamilia wengine | The fact that we have to differentiate my food from that of the rest of the family is already a challenge on our budget on food. It does not only mean cooking of two pots but also the type of food that I am recommended to use is not easily accessible and again it is very expensive compared to that of the rest…” |
| PTIDI18D | Wakati huu ugonjwa ulipoanza ilikuwa kidogo afadhali (mmh) lakini sasa haikuchukua muda nilishangaa tu ikawa haiwezekani tena kabisaa inalegee tu (mmh) yaani hata nifanye nini ile kitu inashindwa haisimami (mmh) yaani nakuambia haiji hata kidogo (mmh) yaani ninamaana siwezi kufanya chochote yaani nipo tu | When this disease started, it was a little better … but then it did not take time….it became completely impossible … whatever I do, that thing [penis] fails … it does not come up … I mean, I cannot do anything [to perform sex]… |
| FMIDI2 | Unavyomwona hapo hana uwezo wa kufanya kitu chochote (mmh) chochote kabisa yaani amekuja kuwa mtu ambaye inabidi tumwangalie kama mtoto mdogo eeh inabidi umtafutie chakula na kumpikia yeye ale tu eeh yaani ni mara chache sana anaweza hata kujipikia chakula chake na napo inakubidi kuhakikisha kila kitu kipo karibu yake basi yeye kazi ni kuweka tu kwenye moto na kuangalia. Kusema kweli huu ungojwa unatisha ni ugonjwa mbaya sana yaani unaweza kumfanya mtu kilema kabisa | ……she is not able to do anything tough …I mean she’s turned into someone we have to look after like a child… you have to find food and cook for her … only on rare occasions can she cook for herself but you need also to make sure everything is closer to her …. Honestly, this is a very strange/bad illness… it can make a person completely disabled |
| FMIDI2 | Kusema ukweli inafikia mahali ambapo huwezi kuwekeza kwenye vitu vingine tu kwa sababu ya kuhitaji kumhudumia huyu mgonjwa (mmh) wakati mwingine unapanga utafanya hivi na vile kwa maendeleo yako lakini bahati mbaya ghafla unakuta hali yake imekuwa sio nzuri hapo unajikuta unalazimika kuacha mipango yako mingine na kumwangalia yeye sababu huwezi kumwacha mgonjwa tu na hali kama hiyo bila huduma na hii inasababisa mwishowe unakuta pesa yote inaishia kwenye huduma zake tu hamna kingine kinaendelea | Honestly, you get to a point where you completely fail to invest in other things,…simply because of the need to take care of this patient…..sometimes you plan that you will do this and that for your own development but unluckily, you suddenly find her condition is not good …then you become forced to stop your other plans and take care of her because you cannot leave the patient just like that… as a result, you find that ultimately all the money is going towards her care… |
| PTIDI119D | Ugonjwa huu umetuletea umasikini mkubwa kwa kweli (mmh) kipindi nilipougua sana ilibidi mke wangu aache biashara zake akae aniuguze (mmh) na kwa sababu ya hali halisi ya kiuchumi iliyokuwepo ilibidi atumie ule mtaji kulipia gharama za madawa na mahitaji mengine (mmh) na kuanzia pale hajaweza tena kurudi kwenye zile biashara maana mtaji umekuwa mgumu (sawa) ndio maana mimi nasema kisukari kimetuletea umasikini na hakuna matumaini yoyote kwamba hii hali itabadilika sababu ugonjwa ndio huu na kupona hauponi | ……..when I was very sick my wife had to stop her business to take care of me…and because of the real situation she used the capital to pay for the bills…. since then she has never been able to go back … That is why I say this Kisukari has brought poverty upon us and there is no hope that this situation will change….” |
| PTIDI7D | Unajua wakati mwingine najisikia vibaya kuomba msaada kwa watu. Kila mara naomba msaada kwa bint yangu na mume wangu ingawa wao hawaonyeshi kuchukia kunisaidia lakini mimi mwenyewe najisikia vibaya maana najiona kama nimekuwa mzigo kwao | …..sometimes I feel sad, asking help from others. I am always asking help from my husband and daughter, and even though they don’t seem to mind helping me, I feel dejected. I feel like am becoming a burden on them… |
| PTIDI3D | Nimwmweleza kila mtu kwenye familia juu ya hii hali yangu na jinsi inavyonipelekesha maana wanahitaji kunichukulia kama nilivyo (mmh) ninakasirika haraka sana na wakati mwingine nashindwa hata kujizuia mwenyewe yaani (mmh) lakini namshukuru Mungu wamenielewa na wananivumilia | I told everyone in the family about my condition…They need to take me as I am, I get angry faster now and sometimes I fail to control myself …I am thankful to God that they understood and are tolerating my condition…. |
| PTIDI5D | changamoto kubwa ninayoiona ni kuzoea hicho chakula cha kisukari (mmh) mimi mwenywe nina sukari na BP na niliashauriwa niache kula chakula chenye chumvi na sukari kwa hiyo sasa unaweza kuona ni kitu ambacho sikuwahi kukiwaza (mmh) lakini sasa sikuwa na la kufanya inabidi kufuata ushauri (mmh) mwanzoni ilikuwa ngumu sana kwa kweli, sikuweza kabisa kufuatilia na matokeo yake nilikuwa mgonjwa wakati wote nipo tu kitandani hii sukari ilikuwa haitulii na BP ilikuwa inapanda kila wakati yaani ilikuwa ni shida sana | …the main challenge I see is getting used to the food …myself, I have diabetes and BP. I was advised to stop salted and sugary foods …so you can imagine this is something that I had never thought about… but I had no choice……at first it was really difficult; I could not manage it and as a consequence I was always sick … the sugar was very unstable and the BP was rising all the time… |
